# Supplementary material for: Self-Reported Medication Adherence Measured with Morisky Scales in Rare Disease Patients: A Systematic Review and Meta-Analysis
Source: Healthcare (Basel). 2023 May 31;11(11):1609. doi: 10.3390/healthcare11111609 (PMC10253179; doi:10.3390/healthcare11111609)
Supplement: Supplementary file 1 [file healthcare-11-01609-s001.zip › healthcare-2327421-supplementary.pdf]

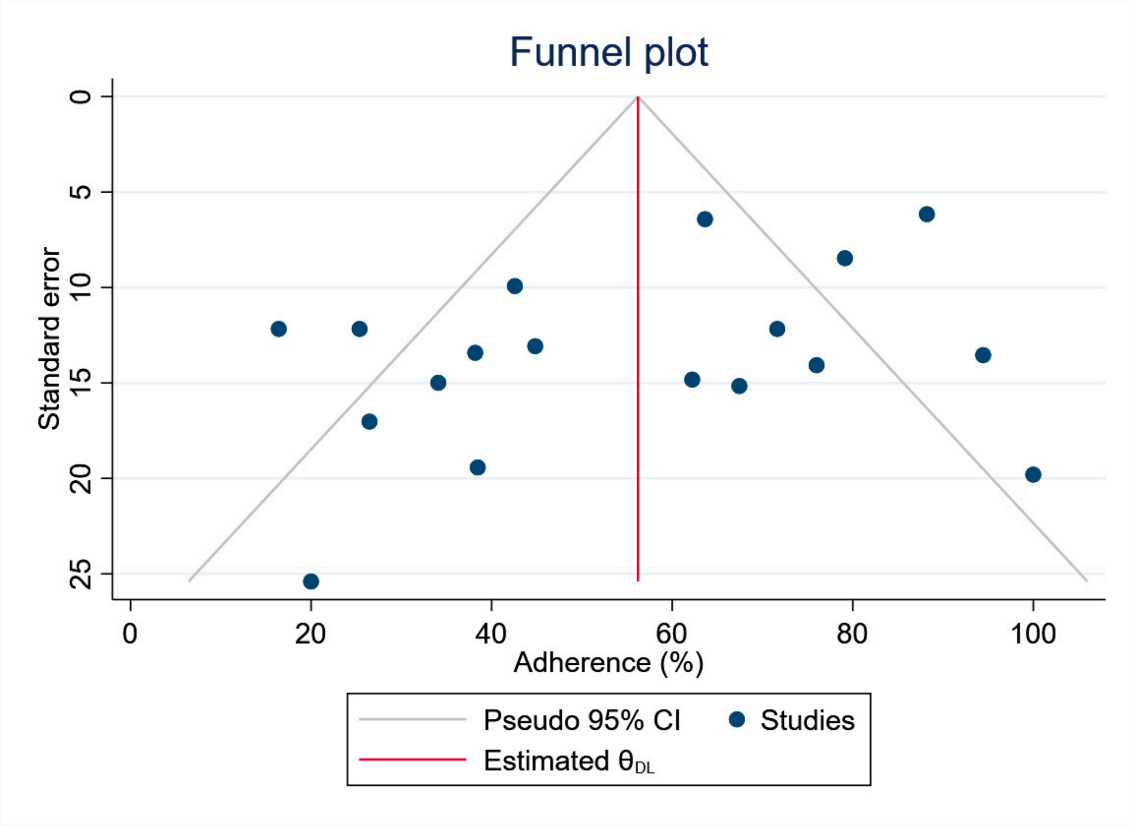

Figure S1. Funnel plot.

Table S1. Search strategy.

Databases searched until 08th December 2022.
Strategy for Web of Science:<br>Final search: TS=((("Morisky Medication Adherence Scale") OR ("MMAS-4") OR ("MMAS-8") OR (“Morisky Green Levine”) OR ("Morisky Green Levine Medication Adherence Scale") OR ("Medication Adherence Questionnaire")) AND (("rare diseases") OR ("narcolepsy") OR ("Primary biliary cholangitis") OR ("fabry disease") OR ("cystic fibrosis") OR (“hemophilia A”) OR (“hemophilia B”) OR ("Sickle Cell Disease") OR ("Myasthenia Gravis") OR ("Idiopathic Pulmonary Fibrosis") OR ("Pulmonary arterial hypertension") OR ("Wilson's disease"))) (No limitations were used).
**Results: 17**
Strategy for PubMed: ("Morisky Medication Adherence Scale"[All Fields] OR "MMAS-4"[All Fields] OR "MMAS-8"[All Fields] OR "Morisky Green Levine Medication Adherence Scale"[All Fields] OR "Morisky Green Levine”[All Fields] OR "Medication Adherence Questionnaire"[All Fields]) AND ("rare diseases"[All Fields] OR "narcolepsy"[All Fields] OR "Primary biliary cholangitis"[All Fields] OR "fabry disease"[All Fields] OR "cystic fibrosis"[All Fields] OR "hemophilia A"[All Fields] OR "hemophilia B"[All Fields] OR "Sickle Cell Disease"[All Fields] OR "Myasthenia Gravis"[All Fields] OR "Idiopathic Pulmonary Fibrosis"[All Fields] OR "Pulmonary arterial hypertension"[All Fields] OR "Wilson's disease"[All Fields]) (No limitations were used).
**Results: 12**
Strategy for SCOPUS: (("Morisky Medication Adherence Scale") OR ("MMAS-4") OR ("MMAS-8") OR ("Morisky Green Levine Medication Adherence Scale") OR ("Morisky Green Levine”) OR ("Medication Adherence Questionnaire")) AND (("rare diseases") OR ("narcolepsy") OR ("Primary biliary cholangitis") OR ("fabry disease") OR ("cystic fibrosis") OR ("hemophilia A") OR ("hemophilia B") OR ("Sickle Cell Disease") OR ("Myasthenia Gravis") OR ("Idiopathic Pulmonary Fibrosis") OR ("Pulmonary arterial hypertension") OR ("Wilson disease")) (No limitations were used).
**Results: 21**
Strategy for Cochrane: ((("Morisky Medication Adherence Scale") OR ("MMAS-4") OR ("MMAS-8") OR ("Morisky Green Levine Medication Adherence Scale") OR (“Morisky Green Levine”) OR ("Medication Adherence Questionnaire")) AND (("rare diseases") OR ("narcolepsy") OR ("Primary biliary cholangitis") OR ("fabry disease") OR ("cystic fibrosis") OR ("hemophilia A") OR ("hemophilia B") OR ("Sickle Cell Disease") OR ("Myasthenia Gravis") OR ("Idiopathic Pulmonary Fibrosis") OR ("Pulmonary arterial hypertension") OR ("Wilson disease"))):ti,ab,kw
**Results: 4**

Table S2. Risk of bias.

Reference	1	2	3	4	5	6	7	8	9	Total score	Summary on the overall risk of study bias
Aşiret et al. (2021)	1	0	1	0	0	0	0	0	0	2	Low risk
Bacci et al. (2019)	0	0	0	0	0	0	0	0	0	0	Low risk
Badawi et al. (2019)	1	0	1	0	0	0	0	0	0	2	Low risk
Dwyer et al. (2016)	1	0	0	0	0	0	0	0	0	1	Low risk
Dzemaili et al. (2017)	0	0	0	0	0	0	0	0	0	0	Low risk
Galadanci et al. (2015)	1	0	0	0	1	0	0	0	0	2	Low risk
Grady et al. (2018)	1	0	0	0	0	0	0	0	0	1	Low risk
Idiázquez et al. (2018)	1	0	1	0	0	0	0	0	0	2	Low risk
Introna et al. (2018)	1	0	1	0	0	0	0	0	0	2	Low risk
Jacquelet et al. (2021)	0	0	0	0	0	0	0	0	0	0	Low risk
Karaca et al. (2022)	1	0	1	0	0	0	0	0	0	2	Low risk
Knudsen et al. (2016)	1	0	0	0	0	0	0	1	0	2	Low risk
Lamiani et al. (2015)	0	0	1	0	0	0	0	0	0	1	Low risk
Raji et al. (2016)	1	0	1	0	0	0	0	0	0	2	Low risk
Thuret et al. (2009)	0	0	1	0	0	0	0	0	0	1	Low risk
Treadwell et al. (2005)	1	0	1	0	1	0	0	0	0	3	Low risk
Viswanathan et al. (2015)	1	0	1	0	1	0	0	0	0	3	Low risk
Vitturi et al. (2019)	1	0	1	0	0	0	0	0	0	2	Low risk
